# Clueless, a protein required for mitochondrial function, interacts with the PINK1-Parkin complex in *Drosophila*

**DOI:** 10.1242/dmm.019208

**Published:** 2015-06-01

**Authors:** Aditya Sen, Sreehari Kalvakuri, Rolf Bodmer, Rachel T. Cox

**Affiliations:** ^1^ Department of Biochemistry and Molecular Biology, 4301 Jones Bridge Road, Uniformed Services University, Bethesda, MD 20814, USA; ^2^ Sanford-Burnham Medical Research Institute, 10901 North Torrey Pines Road, La Jolla, CA 92037, USA

**Keywords:** Clueless, Mitochondria, TOM20, PINK1, Parkin

## Abstract

Loss of mitochondrial function often leads to neurodegeneration and is thought to be one of the underlying causes of neurodegenerative diseases such as Parkinson's disease (PD). However, the precise events linking mitochondrial dysfunction to neuronal death remain elusive. PTEN-induced putative kinase 1 (PINK1) and Parkin (Park), either of which, when mutated, are responsible for early-onset PD, mark individual mitochondria for destruction at the mitochondrial outer membrane. The specific molecular pathways that regulate signaling between the nucleus and mitochondria to sense mitochondrial dysfunction under normal physiological conditions are not well understood. Here, we show that *Drosophila* Clueless (Clu), a highly conserved protein required for normal mitochondrial function, can associate with Translocase of the outer membrane (TOM) 20, Porin and PINK1, and is thus located at the mitochondrial outer membrane. Previously, we found that *clu* genetically interacts with *park* in *Drosophila* female germ cells. Here, we show that *clu* also genetically interacts with *PINK1*, and our epistasis analysis places *clu* downstream of *PINK1* and upstream of *park*. In addition, Clu forms a complex with PINK1 and Park, further supporting that Clu links mitochondrial function with the PINK1-Park pathway. Lack of Clu causes PINK1 and Park to interact with each other, and *clu* mutants have decreased mitochondrial protein levels, suggesting that Clu can act as a negative regulator of the PINK1-Park pathway. Taken together, these results suggest that Clu directly modulates mitochondrial function, and that Clu's function contributes to the PINK1-Park pathway of mitochondrial quality control.

## Abstract (precis)

**Summary:** The protein Clueless is crucial for mitochondrial function and can interact genetically and physically with the PINK1-Parkin mitophagy complex.

## INTRODUCTION

Mitochondrial function is intimately linked to cellular health. These organelles provide the majority of ATP for the cell in addition to being the sites for major metabolic pathways such as fatty acid β-oxidation and heme biosynthesis. In addition, mitochondria are crucial for apoptosis, and they can irreparably damage the cell via oxidation when their biochemistry is abnormally altered. Given these many roles, tissues and cell types with high energy demands, such as neurons, are particularly sensitive to changes in mitochondrial function (Chen and Chan, 2009). This is also true for germ cell mitochondria because mitochondria are inherited maternally from the egg's cytoplasm and are thus the sole source of energy for the newly developing embryo (Schon et al*.*, 2012).

Mitochondrial biology is complex owing to the dynamic nature of the organelle and the fact that most of the proteins required for function are encoded in the nucleus. In addition to the metabolites they provide, mitochondria undergo regulated fission, fusion and transport along microtubules (Bereiter-Hahn and Jendrach, 2010; Chan, 2012). Because mitochondria cannot be made *de novo*, and tend to accumulate oxidative damage due to their biochemistry, they are subject to organelle and protein quality-control measures that involve mitochondrial and cytoplasmic proteases, as well as a specialized organelle-specific autophagy called mitophagy (Jin and Youle, 2012; Margineantu et al., 2007; Soubannier et al., 2012). However, the specific molecular signaling pathways between the nucleus and mitochondria that are used to sense which individual mitochondria are damaged during normal cellular homeostasis *in vivo* are not well understood.

We use the *Drosophila* ovary to identify genes regulating mitochondrial function and have characterized mitochondrial dynamics during *Drosophila* oogenesis (Cox and Spradling, 2003). Germ cells contain large numbers of mitochondria that can be visualized at the single organelle level, making this system useful for studying genes that control mitochondrial function.

The gene *clueless* (*clu*) is crucial for mitochondrial localization in germ cells (Cox and Spradling, 2009). Clu has homologs in many different species, and shows 53% amino acid identity to the human homolog, CLUH. The molecular role of Clu is not known. The yeast homolog, Clu1p, was found to interact with the eukaryotic initiation factor 3 (eIF3) complex in yeast and bind mRNA; however, the significance of this is not clear (Mitchell et al., 2012; Vornlocher et al., 1999). CLUH has also been shown to bind mRNA (Gao et al., 2014). Flies mutant for *clu* are weak, uncoordinated, short-lived, and male and female sterile (Cox and Spradling, 2009). Lack of Clu causes a sharp decrease in ATP, increased mitochondrial oxidative damage and changes in mitochondrial ultrastructure (Cox and Spradling, 2009; Sen et al*.*, 2013). Levels of Clu protein are homogeneously high in the cytoplasm and it is also found in large mitochondrially-associated particles. Although Clu clearly has an effect on mitochondria function, whether this is direct or indirect has not yet been established.

Parkin (Park), an E3 ubiquitin ligase, acts with PTEN-induced putative kinase 1 (PINK1) to target mitochondria for mitophagy (Youle and Narendra, 2011). *clu* genetically interacts with *park*, and Clu particles are absent in *park* mutants, indicating that Clu might play a role in Park's mechanism (Cox and Spradling, 2009; Sen et al*.*, 2013). *park* and *PINK1* have been identified as genes that, when mutated, cause early-onset forms of Parkinson's disease (Kitada et al*.*, 1998; Valente et al*.*, 2004). Upon mitochondrial depolarization, PINK1 is stabilized on the mitochondrial outer membrane, recruiting Park, which then goes on to ubiquitinate many surface proteins, thus marking and targeting that mitochondrion for mitophagy (Narendra et al., 2010a, 2008; Sarraf et al., 2013). Before their biochemical interaction was recognized, *PINK1* was placed upstream of *park* in a genetic pathway in *Drosophila* (Clark et al*.*, 2006; Park et al*.*, 2006; Yang et al*.*, 2006). Understanding Park and PINK1's role in mitochondrial quality control has shed light on the neurodegeneration underlying Parkinson's disease (Nuytemans et al*.*, 2010).

**TRANSLATIONAL IMPACT**

**Clinical issue**

Mitochondrial ATP generation is crucial for cell survival, particularly in cells with a high energy demand such as neurons. However, the creation of ATP induces oxidative damage in mitochondria. Consequently, to control mitochondrial quality, cells have developed mechanisms that destroy damaged mitochondrial proteins, and that can target the entire organelle for destruction via a process known as mitophagy. PTEN-induced kinase 1 (PINK1) and Parkin (Park) are two extensively studied proteins that function in mitochondrial quality control. Notably, these two proteins are mutated in inherited forms of Parkinson's disease. Thus, the identification of further proteins involved in PINK1 and Park function is a priority for understanding Parkinson's disease.

**Results**

Clueless (Clu) is a large, highly conserved protein that is required for normal mitochondrial function; however, where it exerts its function is unclear. Here, the authors show that the human homolog of Clu can rescue *Drosophila clu* mutants. Then, working in *Drosophila*, they show that Clu peripherally associates with mitochondria by binding three mitochondrial outer membrane proteins: Translocase of the outer membrane 20 (TOM20), Porin and PINK1. Using epistasis analysis, they demonstrate that *clu* genetically functions downstream of *PINK1* and upstream of *park* in the PINK1-Park pathway in cell culture and *in vivo*. Importantly, they show that Clu forms a complex with PINK1, and also interacts with Park, but only when the mitochondrial membrane potential is lost with the addition of the ionophore CCCP. Finally, the authors report that the expression of several mitochondrial proteins is greatly decreased *in vivo* in *clu* and *PINK1* mutants, but not in *park* mutants.

**Implications and future directions**

Together, these results identify Clu as a newly identified component of the PINK1-Park pathway in *Drosophila* and suggest that Clu functions as a negative regulator of PINK1-Park function. Yeast Clu1 has been shown to be a component of the eIF3 complex and to bind mRNA, and human Clu can bind the mRNA of nuclear-encoded mitochondrial proteins. The authors therefore propose a model in which Clu functions to assist mitochondrial protein import, thereby acting as a sensor for mitochondrial function. Understanding how a breakdown in mitochondrial function causes Parkinson's disease is imperative, but the specific molecular pathways that sense mitochondrial dysfunction under normal physiological conditions remain unclear. The identification of Clu as a potential sensor for mitochondrial quality advances our understanding of mitochondrial function and might, therefore, lead to a better understanding of Parkinson's disease and other neurodegenerative diseases.

Here, we show that Clu's mitochondrial role is well conserved, because the human homolog, CLUH, can rescue the fly mutant. Clu peripherally associates with mitochondria because it forms a complex with the mitochondrial outer-membrane proteins Porin and Translocase of the outer membrane (TOM) 20, supporting that the loss of mitochondrial function caused by lack of Clu is a direct effect. In addition, we find that *clu* genetically interacts with *PINK1* and, using epistasis, we place *clu* upstream of *park*, but downstream of *PINK1*. Clu forms a complex with PINK1, and is able to interact with Park after the mitochondrial membrane potential is disrupted. Finally, lack of Clu causes PINK1 and Park to interact with each other, as well as causing a decrease in mitochondrial proteins, which suggests that Clu negatively regulates PINK1-Park function. Taken together, these data identify Clu as a mitochondrially-associated protein that plays a direct role in maintaining mitochondrial function and that binds TOM20, and support a role for Clu linking mitochondrial function to the PINK1-Park pathway.

## RESULTS

### Expressing human *CLUH* can rescue *clu* mutant flies

**Fig. 1.**
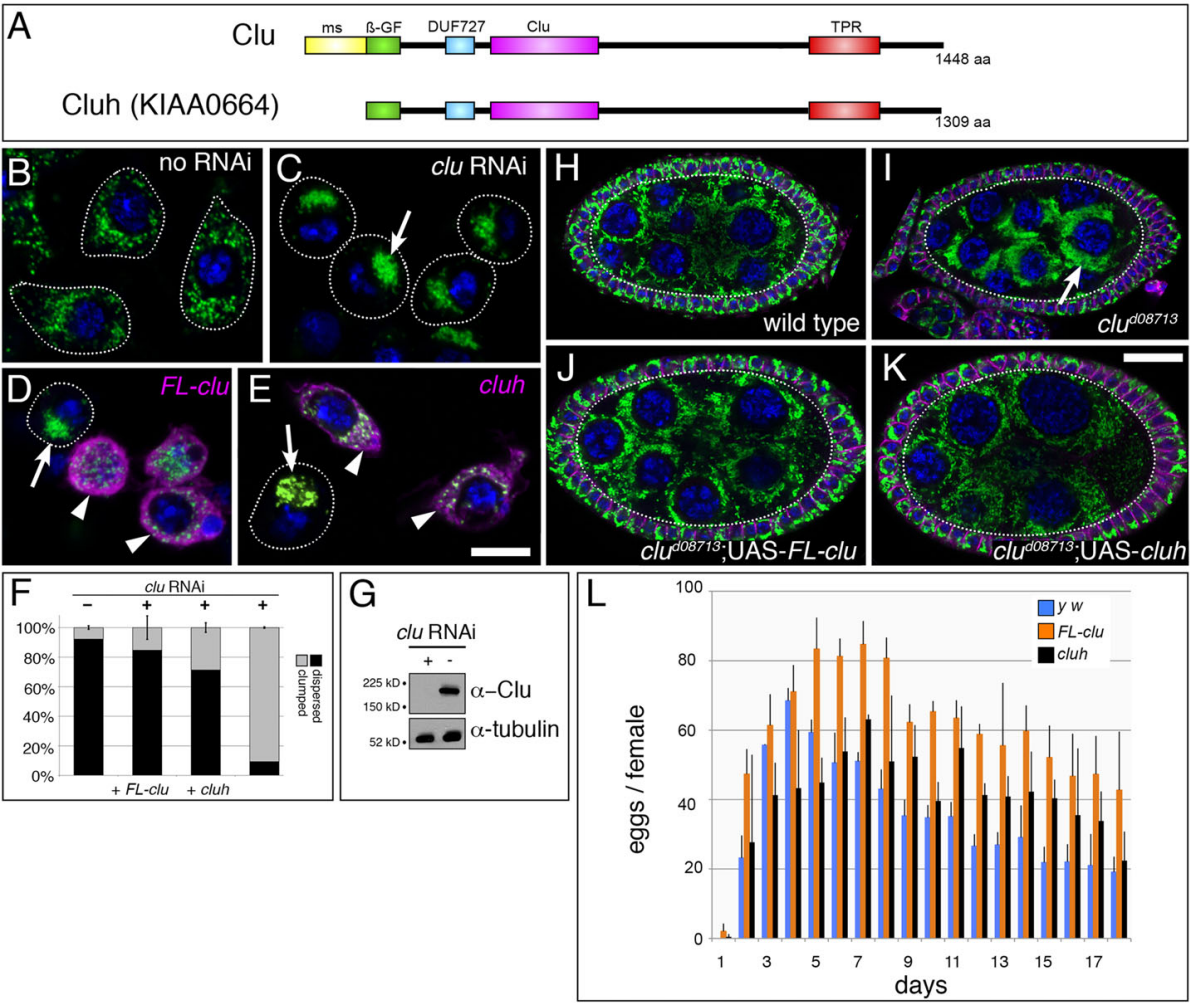
**The human homolog of *clu*, *CLUH*, can rescue *Drosophila clu* mutant phenotypes.** (A) Schematic of *Drosophila* and human Clu/CLUH. *In silico* analysis predicts several domains. Clu, Clu domain; TPR, tetratricopeptide repeats; DUF, domain of unknown function; β-GF, beta grasp fold; ms, melanogaster specific. (B-E) S2R+ cells normally have dispersed mitochondria (B, green). Upon *clu* RNAi treatment, mitochondria become tightly clumped (C, green, arrow). The mitochondrial clumping (D,E, arrows) is rescued by expressing either *Drosophila* full-length Clu (D, magenta, arrowheads) or CLUH (E, magenta, arrowheads). Dotted lines outline cells. (F) Quantification of B-E. See Materials and Methods for details. (G) A western blot demonstrating that *clu* RNAi treatment effectively eliminates Clu protein. (H-K) Female germ cells labeled with mitochondria (green) and membranes (magenta) to outline and mark the somatic cells. Mitochondria in wild-type germ cells (H, green) are dispersed in the germplasm (dotted line). Germ cells lacking Clu (I, dotted line) have clumped mitochondria (green, arrow). Overexpressing either *Drosophila* full-length Clu (J) or CLUH (K) disperses mitochondria compared with in the *clu* mutant. (L) Expressing Clu or CLUH in a *clu*-null background completely rescues the sterility of *clu*-null mutant females. Green=α-CVA, magenta=α-GFP (D,E), α-1B1 (hu li tai shao, adducin-like protein) (H-K), blue=DAPI. Scale bars: 10 μm in E for B-E, in K for H-K.

*Drosophila* Clu and human CLUH share 53% amino acid identity throughout their lengths, with particularly high (85%) identity between their Clu domains (Fig. 1A) (Cox and Spradling, 2009). Using homology searches and online analysis, we have identified, in addition to the tetratricopeptide repeat domain (TPR) (Zhu et al., 1997), two other potential domains, DUF 727 and a beta-Grasp Fold (β-GF), in these proteins (Fig. 1A). *Drosophila* Clu has an additional 100 amino acids at the N-terminus that are not found in CLUH. This N-terminal domain is specific to the *Drosophila melanogaster* and *obscura* groups, but degenerates in species further away. To determine whether CLUH can rescue the phenotypes associated with loss of Clu, we expressed CLUH in S2R+ cells and *Drosophila*. Ninety percent of S2R+ cells had evenly dispersed mitochondria that were fragmented (Fig. 1B,F). After treating the cells with *clu* RNAi to knock down Clu protein (Fig. 1G), mitochondria became mislocalized and clumped together in one to three clusters in the cell (Fig. 1C, arrow, 1F). This clumping phenotype can be rescued by transfecting the *clu*-RNAi-treated cells with either full-length *clu* (Fig. 1D, magenta, arrowheads) or *CLUH* (Fig. 1E, magenta, arrowheads). To test whether *CLUH* can rescue phenotypes associated with *clu*-null mutant flies, we overexpressed full-length *Drosophila clu* (*FL*-*clu*) and *CLUH* in the *clu^d08713^*-null background using the GAL4/UAS system (supplementary material Fig. S1A) (Brand and Perrimon, 1993). In the ovary, germ cell mitochondria were evenly dispersed in wild type (Fig. 1H) (Cox and Spradling, 2003). In *clu^d08713^* mutant germ cells, mitochondria were mislocalized and highly clustered (Fig. 1I, arrow) (Cox and Spradling, 2009). Upon ubiquitously overexpressing *FL*-*clu* or *CLUH* using *daughterless* (*da*) GAL4, we found that the mitochondria were much more dispersed and had a more wild-type pattern of distribution (Fig. 1J,K). *clu^d08713^* mutant females are completely sterile and never lay eggs (Cox and Spradling, 2009). However, expressing *FL*-*clu* or *CLUH* using *da* GAL4 rescued the egg-laying ability of females (Fig. 1L), as well as their ability to climb (supplementary material Fig. S1B). These results show that the mitochondrial mislocalization phenotype, resulting sterility, and locomotion defects in *clu^d08713^* mutants are due specifically to loss of *clu*, and that the human homolog *CLUH* is able to use the *Drosophila* machinery to rescue these deficits.

### Clu peripherally associates with mitochondria

**Fig. 2.**
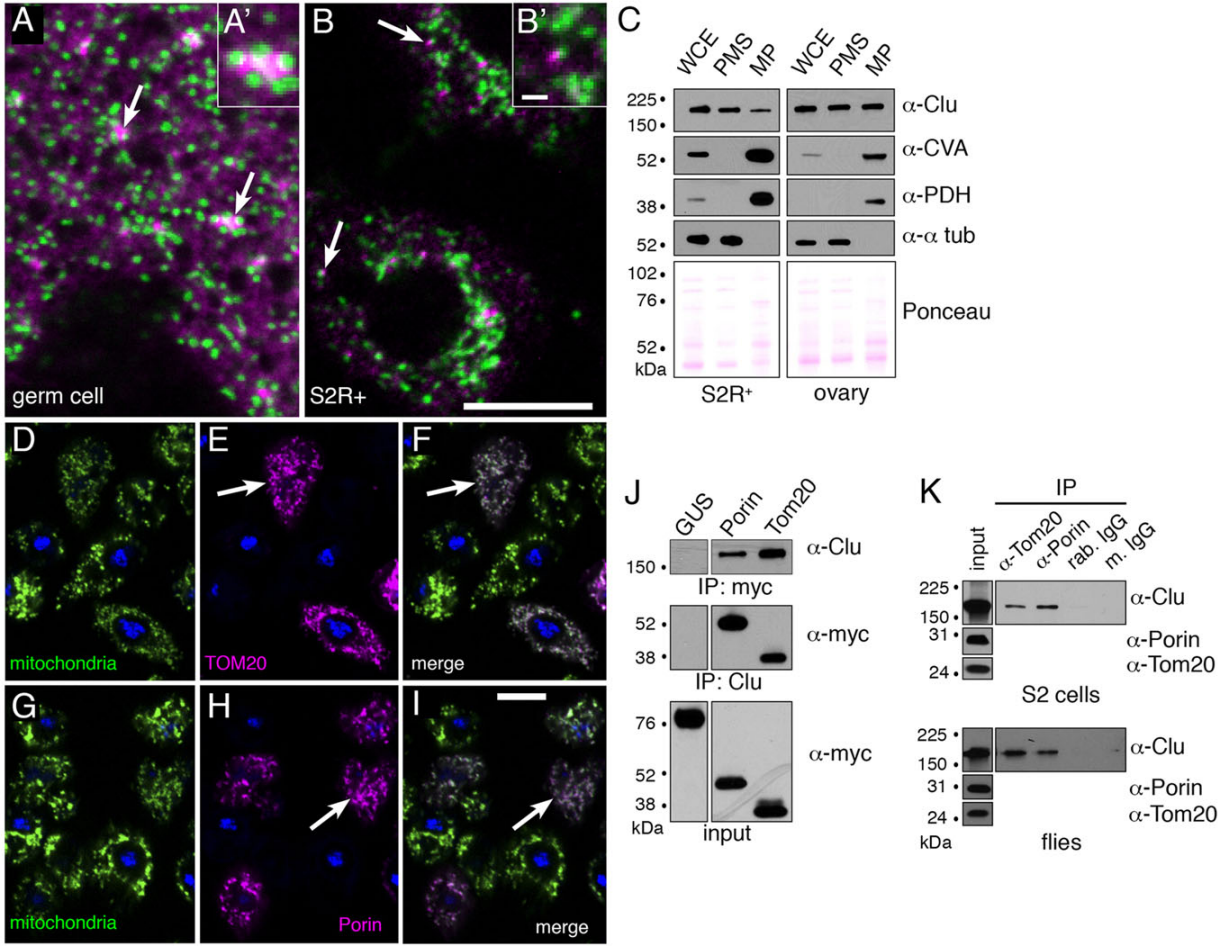
**Clu is a peripheral mitochondrial protein.** (A,B) Germ cells (A) and S2R+ cells (B) labeled for mitochondria (green) and Clu (magenta). Clu protein levels are high in the cytoplasm, and Clu forms mitochondrial-associated particles. (A) Germ cells have homogeneous cytoplasmic Clu, as well as prominent Clu particles (A, arrows) that are closely associated with mitochondria (green, A′). (B) Mitochondrially-associated Clu particles (arrows, B,B′) are also seen in S2R+ cells. (C) Cell fractionation of S2R+ and ovary extract shows that Clu is found in both the post-mitochondrial supernatant (PMS) and mitochondrial pellet (MP). WCE, whole cell extract; CVA, Complex V; PDH, pyruvate dehydrogenase; tub, tubulin. (D-I) S2R+ cells transfected with myc-tagged TOM20 (D-F, magenta, arrows) or myc-tagged Porin (G-I, magenta, arrows) colocalize with mitochondria (green). (J) A western blot of S2R+ cells transfected with myc-tagged Porin, TOM20 and GUS (as a negative control). Performing reciprocal co-immunoprecipitations shows that Clu is in a complex with Porin and TOM20. (K) Clu is found in a complex with endogenous TOM20 and Porin in S2R+ cells and fly extract. IPs using rabbit (rab.) IgG and mouse (m.) IgG were performed as controls for the anti-TOM20 and -Porin antibodies, respectively. GUS, myc-tagged plant glucuronidase. Scale bars: 10 μm in B for A,B, and in I for D-I; 1 μm in B′ for A′,B′.

Clu protein is highly abundant in the cytoplasm of female germ cells. Immunofluorescence shows Clu at homogeneously high levels in the cytoplasm, as well as in particles (Fig. 2A, arrows), which are always tightly associated with germ cell mitochondria (Fig. 2A,A′, arrows) (Cox and Spradling, 2009). Mitochondrially-associated Clu particles are also found in the cytoplasm of S2R+ cells (Fig. 2B,B′, arrows). To further investigate Clu's potential association with mitochondria, we fractionated S2R+ cells and ovaries, and found that Clu is present in the mitochondrial pellet, as well as the post-mitochondrial supernatant (Fig. 2C).

Loss of Clu results in mitochondrial oxidative damage and a decrease in ATP production (Sen et al*.*, 2013). As a starting point to elucidate Clu's molecular function in the cell, we used immunoprecipitation (IP) and performed mass spectrometry on several discrete gel bands that differed between control and Clu IPs in order to identify potential Clu protein partners (data not shown). Using this approach, we identified Porin [also known as Voltage dependent anion channel (VDAC)], as well as TOM20. Porin is an integral mitochondrial outer-membrane protein that is a target of Park's E3 ubiquitin ligase activity (Geisler et al*.*, 2010; Narendra et al*.*, 2010a). TOM20 has a transmembrane domain spanning the mitochondrial outer membrane and acts as one of the receptors for protein import into mitochondria. PINK1 physically associates with the TOM complex, and can directly bind TOM20 (Lazarou et al*.*, 2012). S2R+ cells transfected with myc-tagged TOM20 and Porin showed mitochondrial localization, as expected (Fig. 2D-I, arrows). To confirm our IP and mass spectrometry results, we performed reciprocal co-IPs between endogenous Clu and myc-tagged TOM20 and Porin, and found that both can form a complex with Clu (Fig. 2J). We also found that TOM20 can pull down Clu in fly extract from flies overexpressing UAS-TOM20-myc (supplementary material Fig. S2A). These interactions could be direct or indirect. To ensure that the TOM20 and Porin interactions are not due to the presence of small fragments of mitochondrial outer membrane in the extract, we repeated the co-IPs with S2R+ extract in additional detergent to efficiently solubilize the mitochondria outer membrane and, after high-speed centrifugation, found that the interactions were still present (supplementary material Fig. S2A,B). To rule out that these interactions are due to overexpression artifacts, we used commercially available anti-TOM20 and -Porin antibody and found that they can pull down Clu from both S2R+ and fly extract (Fig. 2K). These results indicate that Clu is present at the mitochondrial outer membrane and peripherally associates with mitochondria. This is the first evidence that Clu's effect on mitochondrial function is direct.

### *PINK1* genetically interacts with *clu*

To better understand what function Clu plays at the mitochondrial outer membrane and to determine its role in mitochondrial function, we took a candidate gene approach to characterize additional interactors. Many phenotypes associated with *clu* mutants are shared with those of *park* and *PINK1* mutant flies, both of which are essential for mitochondrial function, although *clu* mutants are in general much sicker (Clark et al., 2006; Cox and Spradling, 2009; Greene et al., 2003; Park et al., 2006; Yang et al., 2006). PINK1 is targeted to mitochondria, where it is degraded under normal circumstances. When mitochondria lose their membrane potential, for example upon treatment with the ionophore carbonyl cyanide m-chlorophenyl hydrazone (CCCP), PINK1 becomes stabilized on the mitochondrial outer membrane and is presented to the cytoplasm (Lin and Kang, 2008; Matsuda et al*.*, 2010; Narendra et al*.*, 2010b). Once there, PINK1 recruits Park to the mitochondrial outer membrane by an unknown mechanism. *park* mutant germ cells have severely clumped mitochondria that are frequently very long and fused (Cox and Spradling, 2009). Females with different *park*-null allelic combinations have greatly reduced rates of egg laying (supplementary material Fig. S3).

**Fig. 3.**
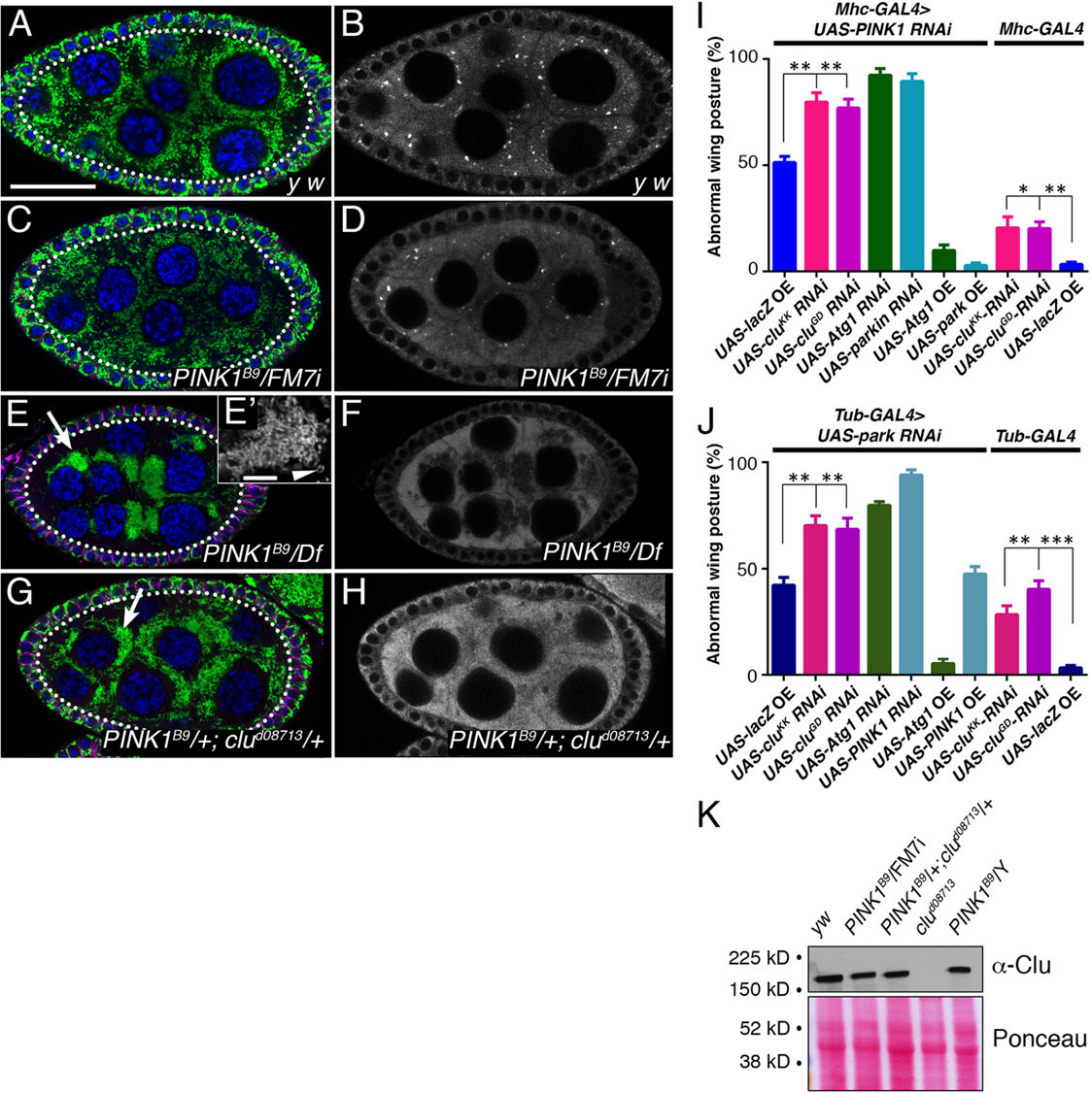
***PINK1* genetically interacts with *clu*.** (A-D) Wild-type (A,B) and *PINK1^B9^*/FM7i (C,D) germ cells (dotted line) contain evenly dispersed mitochondria (green, A,C) and robust Clu particles (white, B,D). Membranes (magenta) are labeled to outline and distinguish the somatic follicle cells. (E,F) Germ cells lacking *PINK1^B9^* have clumped, mislocalized mitochondria (E, arrow) that can be doughnut shaped (E′, arrowhead) and lack Clu particles (F). (G,H) Germ cells that are doubly heterozygous for *clu* and *PINK1^B9^* have mislocalized, clumped mitochondria (G, arrow) and lack Clu particles (H). (I) Simultaneously expressing *PINK1* and *clu* RNAi in flight muscle significantly increases the number of flies with abnormal wing posture compared to controls. (J) Simultaneously expressing both *park* and *clu* RNAi in flight muscle gives the same result. (K) Clu levels are approximately the same in *PINK1* heterozygotes and *PINK1*/+; *clu*/+ transheterozygotes. Green=α-CVA, magenta=α-1B1 (A,C,E,G), blue=DAPI (A,C,E,G), white=α-CVA (E′) and α-Clu (B,D,F,H). Scale bars: 5 μm in E′ for E′; 30 μm in A for A-H.

If the phenotype caused by the lack of two genes deviates from the expected combined individual phenotypes, these two genes are said to interact (Mani et al., 2008; Phillips, 2008). We previously showed that *clu* and *park* double-heterozygous flies have abnormal mitochondrial clustering in the germ cells, indicating that there is a genetic interaction between the two (Cox and Spradling, 2009). We found a similar genetic interaction between *clu* and *PINK1*, further supporting that *clu* genetically functions in the same pathway (Fig. 3). For most of oogenesis, mitochondria in germ cells are found evenly dispersed throughout the cytoplasm (Fig. 3A,C) (Cox and Spradling, 2003). In germ cells lacking *PINK1*, mitochondria become mislocalized and form large clumps (Fig. 3E, arrow). The presence of doughnut-shaped mitochondria suggests that these clumps might consist of abnormally fused mitochondria (Fig. 3E′, arrowhead). These phenotypes are consistent with the mitochondrial morphology in *park* mutant female germ cells (Cox and Spradling, 2009). Both *PINK1* and *park* mutant males have defects in mitochondrial fission and fusion, which was first recognized by their inability to properly form the male germ cell mitochondrial derivative called the Nebenkern (Clark et al., 2006; Riparbelli and Callaini, 2007; Hales and Fuller, 1997). This is in contrast to the clumped mitochondria in *clu* mutants, which do not show defects in fission and/or fusion, as viewed by transmission electron microscopy (TEM) and by analyzing Nebenkern development (Cox and Spradling, 2009). *clu* mutant males are sterile, but their sterility is due to defects that occur later in spermatogenesis, unlike *PINK1* and *park* mutants. Whereas mitochondria remained dispersed in *PINK1* (Fig. 3C) and *clu* heterozygotes, double heterozygotes showed abnormal clusters of mitochondria (Fig. 3G, arrow). These results indicate that *clu* can interact genetically with *PINK1*, and support that the genetic interaction seen between *clu* and *park* is specific.

*clu* mutant flies have wings that are paralyzed up or down and show muscle degeneration, similar to *park-* and *PINK1*-null mutant adults (Clark et al., 2006; Cox and Spradling, 2009; Greene et al., 2003; Park et al., 2006; Yang et al., 2006). Reducing *PINK1* or *park* expression in flight muscle using RNAi mimicked this effect, and we found that expressing two different *clu* RNAi lines in flight muscle or ubiquitously causes an increase in abnormal wing posture (Fig. 3I,J). There is evidence that double-RNAi can act in a similar manner as examining genetic interactions using traditional mutants and, in fact, double-RNAi seems to act in a manner analogous to a synthetic interaction, and can thus identify genes that act in the same pathway (Bakal, 2011; Horn et al., 2011; Marinho et al., 2013). Along these lines, we found that expressing RNAi simultaneously for both *PINK1* and *park*, two genes known to have a genetic interaction, causes an increase in abnormal wing posture (Fig. 3I,J). In addition, co-expressing *Atg1* RNAi with either *PINK1* or *park* also caused an increase in abnormal wing posture, as has been previously demonstrated (Liu and Lu, 2010). Atg1 is required for autophagy and functions downstream of the PINK1-Park pathway (Scott et al., 2007). Co-expressing *clu*- and either *PINK1*- (Fig. 3I) or *park*- (Fig. 3J) RNAi also increased the number of flies with droopy wings, further supporting that there is a genetic interaction between *clu* and *PINK1* and *park*.

Clu protein was found to be highly abundant in the cytoplasm, particularly in germ cells. Clu also formed particles of various sizes in both germ cells and other cell types (Fig. 2A,B) (Cox and Spradling, 2009; Sen et al*.*, 2013). We previously showed that germ cells that are mutant for *park* no longer have particles even though Clu levels remain the same (Cox and Spradling, 2009). Similarly, we found here that germ cells lacking *PINK1* no longer have Clu particles (Fig. 3F). Interestingly, we found that *clu*-*PINK1* heterozygotes not only mislocalize mitochondria, they also no longer form Clu particles (Fig. 3H). Clu protein levels were not reduced; thus, lack of Clu particles in *PINK1* and *park* mutants is due to a change in Clu particle dynamics, and not simply a reduction in the total amount of Clu protein (Fig. 3K). Together, these data lend further support to the notion that Clu has a role in the PINK1-Park pathway involving mitochondrial function, and that lack of either PINK1 or Park causes a change in Clu's subcellular dynamics.

### *clu* functions genetically in between *PINK1* and *park*

**Fig. 4.**
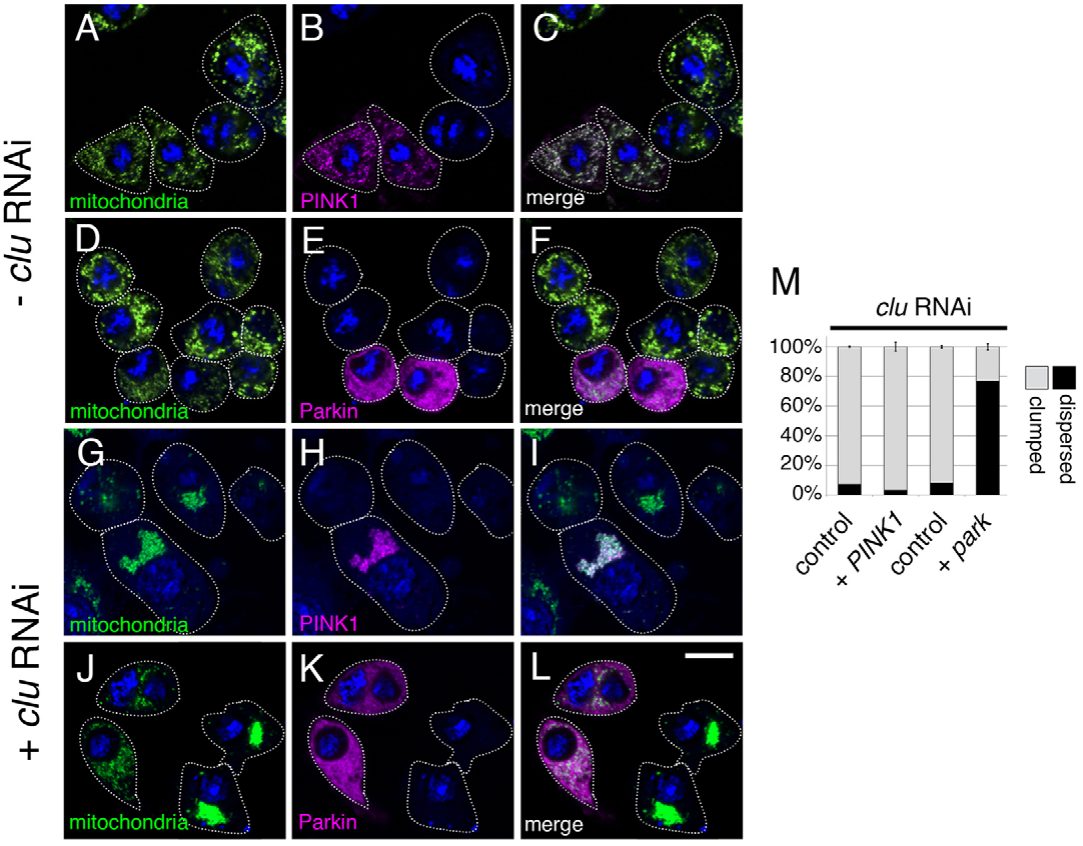
***clu* acts upstream of *park*, and downstream of *PINK1*, in S2R+ cells.** Control (A-F) and *clu*-RNAi-treated (G-L) S2R+ cells transfected with myc-tagged PINK1 (A-C,G-I) or myc-tagged Park (D-F,J-L). Cells transfected with PINK1 show that PINK1 (magenta, B,C,H,I) colocalizes as expected with mitochondria (green, A,C,G,I). Cells transfected with Park show high cytoplasmic levels of Park (magenta, E,F,K,L) that does not obviously colocalize with mitochondria (green, D,F,J,L). Overexpressing PINK1 in cells treated with *clu* RNAi does not rescue the mitochondrial mislocalization phenotype (G-I). In contrast, cells treated for *clu* RNAi that overexpress Park have normal mitochondrial distributions (J,L). (M) Quantification of G-L. Details are in the Materials and Methods. Green=α-CVA, magenta=α-myc, blue=DAPI. Scale bar: 10 μm in L for A-L.

*PINK1* and *park* have been shown to act in the same genetic pathway using epistasis analysis and overexpression in *Drosophila*, with *PINK1* acting upstream of *park* (Clark et al*.*, 2006; Park et al*.*, 2006). Overexpressing *park* in a *PINK1*-null background can rescue some of the phenotypes associated with loss of *PINK1*, such as ultrastructural defects in muscle and male germ cell mitochondria (Clark et al., 2006; Park et al., 2006). To examine whether overexpressing either Park or PINK1 can rescue *clu* mutant phenotypes, we first used S2R+ cells. PINK1 contains an N-terminal mitochondrial targeting sequence. As expected, C-terminally myc-tagged PINK1 transfected into S2R+ cells localized to mitochondria (Fig. 4B,C) (Whitworth et al*.*, 2008). Overexpressing myc-tagged Park in S2R+ cells resulted in cytoplasmic localization, also as expected (Fig. 4E,F) (Narendra et al*.*, 2008). Overexpressing PINK1 in cells that have been treated with *clu* RNAi failed to disperse the clumped mitochondria (Fig. 4G,I,M). In contrast, overexpressing Park rescued the mitochondrial mislocalization phenotype, resulting in dispersed mitochondria (Fig. 4J,L,M). This indicates that *clu* function is upstream of *park*, and downstream of *PINK1*.

**Fig. 5.**
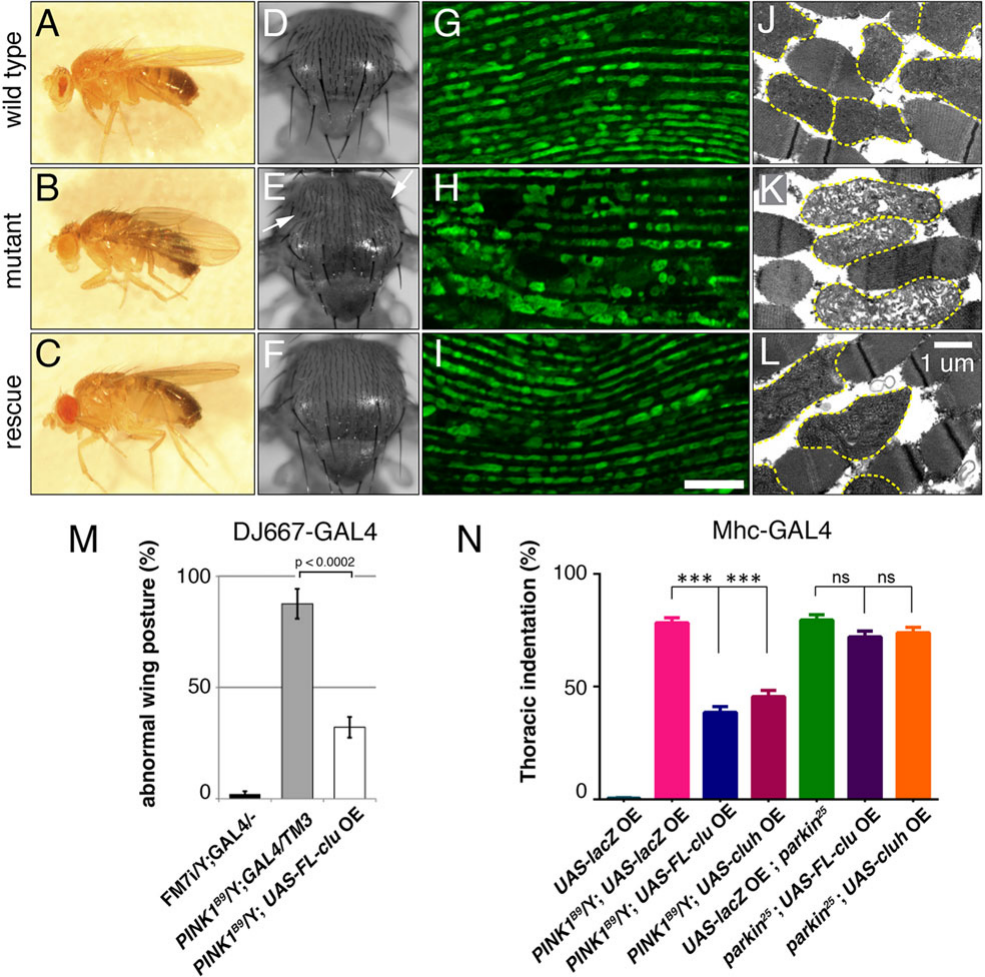
***clu* acts upstream of *park* and downstream of *PINK1* in flies.** (A-M) Overexpressing *clu* using the muscle-expressed DJ667-GAL4 rescues *PINK1* phenotypes. (A-F) FM7i/Y;GAL4/− sibling control flies have normal wing posture (A) and no thoracic indentations (D). *PINK1^B9^*/Y;GAL4/TM3 flies have the expected abnormal wing posture (B) and thoracic indentations (arrows, E). *PINK1^B9^*/Y;GAL4/*FL*-*clu* have greatly improved wing posture and thoraxes (C,F). (G-L) Expressing Clu in a *PINK1*/Y background improves mitochondrial phenotypes. (G-I) Flight muscle immunofluorescence on flight muscle in wild type (G) shows a normal reiterated pattern of mitochondria (green). *PINK1^B9^*/Y; GAL4/TM3 mutant flight muscle shows an irregular pattern of swollen clumped mitochondria (H). Overexpressing Clu rescues the mitochondrial phenotype (I). (J-L) This mitochondrial rescue can be seen at the ultrastructural level using transmission electron microscopy. Mitochondria in *PINK1^B9^*/Y;GAL4/TM3 flight muscle are swollen with a fragmented inner membrane compared to control FM7i/Y; GAL4/− siblings (J versus K, yellow dashed outlines). *PINK1^B9^*/Y;GAL4/*FL*-*clu* overexpressing *clu* exhibit rescued inner mitochondrial membrane (L, yellow dashed outlines). (M) Quantification of A-C. (N) Overexpressing *clu* or *CLUH* in *PINK1*/Y null mutants using Mhc-GAL4 significantly rescues the thoracic indentation phenotype. In contrast, overexpressing *clu* or *CLUH* in a *park*-null mutant has no effect. Green=α-CVA (G-I). Scale bar: 10 μm in I for G-I; 1 μm in L for J-L.

If *clu* functions downstream of *PINK1*, we would expect that *clu* overexpression would be able to rescue *PINK1* mutant phenotypes. To test this, we overexpressed *clu* in a *PINK1* mutant background and examined muscle and mitochondrial phenotypes. *PINK1*/Y mutant males had abnormal wing posture compared with wild-type posture (Fig. 5A versus B). *PINK1*/Y flies overexpressing *FL*-*clu* using a muscle-specific GAL4 driver, DJ667, showed substantial rescue of the abnormal wing posture (Fig. 5C,M) (Seroude et al., 2002). In addition, those rescued flies also had normal thoracic cuticle compared to the indentations found in *PINK1*/Y mutant flies (Fig. 5F versus E, arrows). Not only was the wing posture improved, mitochondrial morphology was also improved in *PINK1*/Y mutants overexpressing *clu*. Immunofluorescence of flight muscle showed that the normal reiterated pattern of mitochondria found in wild-type flies (Fig. 5G) is grossly disrupted in *PINK1*/Y mutants (Fig. 5G versus H). This pattern was rescued upon *FL*-*clu* overexpression (Fig. 5I). The mitochondrial ultrastructure was also rescued, as viewed by TEM (Fig. 5L versus K, yellow dashed outlines). *PINK1*/Y flight muscle mitochondria lacked a mitochondrial inner membrane, whereas rescued mitochondria in *PINK1*/Y flied overexpressing *FL*-*clu* had a much denser inner membrane, similar to wild type (Fig. 5J-L).

Loss of *PINK1* and *park* disrupts flight muscle function to such a great extent that the muscle degenerates, causing the dorsal cuticle on the thorax to cave in (Fig. 5E) (Clark et al., 2006; Cox and Spradling, 2009; Greene et al., 2003; Park et al., 2006; Yang et al., 2006). Using a myosin heavy chain GAL4 (Mhc-GAL4), we found that overexpressing *clu* and *CLUH* in a *PINK1*-null mutant significantly reduces the amount of thoracic indentation (Fig. 5N). In contrast, the frequency of this phenotype in *park*-null mutants did not change upon *FL*-*clu* overexpression (Fig. 5N). Our cell-culture and *in vivo* data strongly support that not only can *clu* function in a common genetic pathway with *PINK1* and *park*, *clu* functions upstream of *park* and downstream of *PINK1*.

### Clu forms a complex with PINK1 and Park

**Fig. 6.**
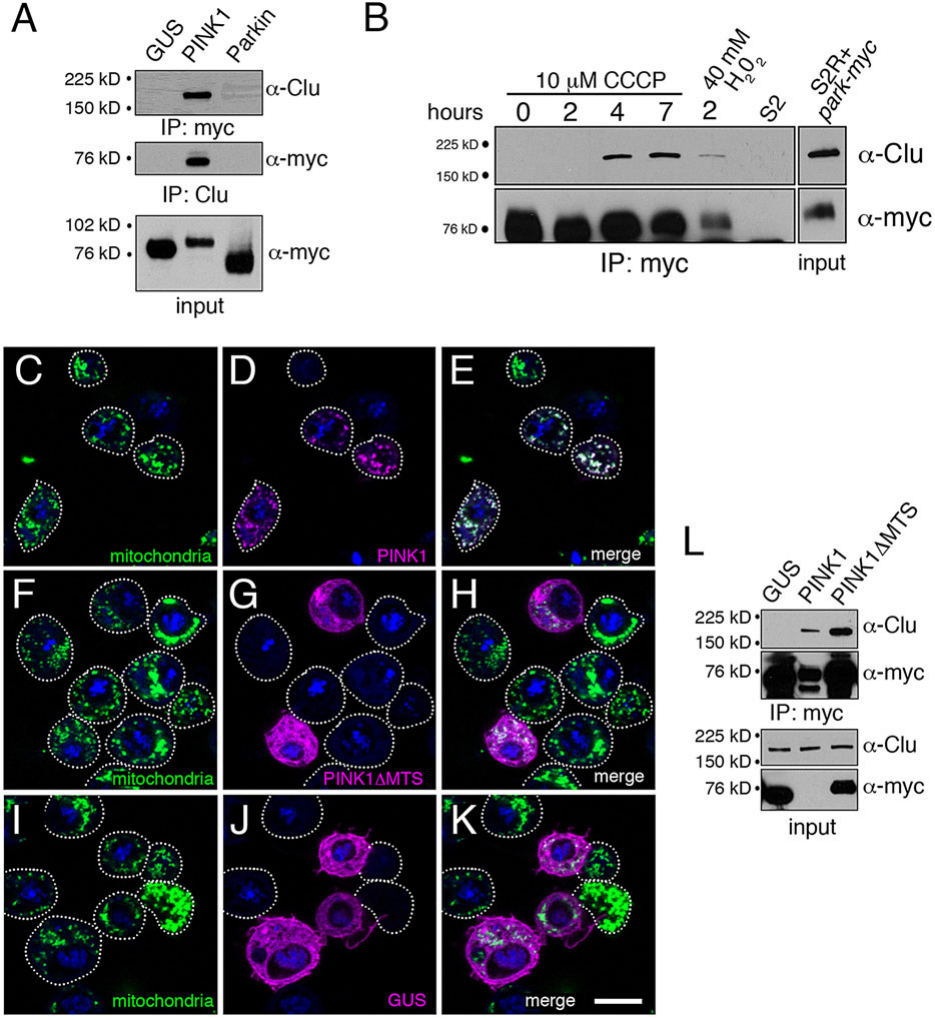
**Clu forms a complex with PINK1 and Park.** (A) A western blot showing extract from S2R+ cells transfected with myc-tagged PINK1 and Park. Performing reciprocal immunoprecipitations shows that Clu is in a complex with PINK1, but not Park, under normal culture conditions. GUS=myc-tagged plant glucuronidase as a negative control, which is from the same experiment and blot as in Fig. 2J. (B) Treating S2R+ cells with CCCP enables Park to immunoprecipitate with Clu. Adding H_2_O_2_ to the cells can also cause an interaction. (C-L) PINK1 lacking the mitochondrial targeting signal (MTS) still interacts with Clu. (C-E) S2R+ cells transfected with PINK1-myc (magenta, D,E) show mitochondrial localization (C,E, green). (F-H) S2R+ cells transfected with PINK1ΔMTS-myc show only cytoplasmic localization (G,H, magenta). (I-K) GUS-myc-transfected cells as a cytoplasmic control. (L) A western blot showing extract from S2R+ cells transfected with myc-PINK1 and myc-PINK1ΔMTS. Both PINK1 and PINK1ΔMTS co-immunoprecipitate with Clu under normal cell culture conditions. Green=α-CVA (C,E,F,H,I,K), magenta=α-myc (D,E,G,H,J,K), blue=DAPI for C-K. Scale bar: 10 μm in K for C-K.

Because we can demonstrate that Clu is present at the mitochondrial membrane, and that *clu* shows a genetic interaction with *PINK1* and *park*, we tested, using reciprocal co-IPs, whether PINK1 or Park can form a complex with Clu. Under the normal conditions for growing and transfecting S2R+ cells, we found Clu and PINK1 in a complex, but not Clu and Park (Fig. 6A). However, upon stressing the cells using CCCP or hydrogen peroxide, Park formed a complex with Clu (Fig. 6B). Park is normally found in the cytoplasm, and is recruited to mitochondria via PINK1 in response to mitochondrial insult and depolarization. Because Clu is in a complex with Park only after treating with CCCP or hydrogen peroxide, Clu must form a complex with Park at the mitochondrial outer membrane and not in the cytoplasm. In contrast, PINK1 and Clu are in a complex under normal conditions. Because PINK1 is normally at low levels in the cell due to degradation, the fact that PINK1 and Clu can form a complex indicates that there must be a small amount of PINK1 at the mitochondrial surface. Our method uses overexpression of PINK1 driven by the actin promoter; thus, PINK1 levels are higher than endogenous levels and might overwhelm the proteases located in the mitochondrial inner membrane and mitochondrial matrix that normally degrade it.

To ensure that the Clu-PINK1 interaction is specific, and not simply due to the presence of mitochondrial outer-membrane fragments, we transfected S2R+ cells with a myc-tagged PINK1 construct lacking the mitochondrial targeting sequence (PINK1ΔMTS) and performed IPs with an increased detergent concentration and after high-speed centrifugation. PINK1ΔMTS-transfected S2R+ cells showed high levels of PINK1ΔMTS in the cytoplasm (Fig. 6G,H), in contrast to the mitochondrial localization seen with full-length PINK1 (Fig. 6D,E). Clu still co-immunoprecipitated with both PINK1 and PINK1ΔMTS under these conditions (Fig. 6L). Note that the levels of PINK1 were much lower than those of PINK1ΔMTS (Fig. 6L, α-myc blots). This is likely because PINK1 is undergoing the normal degradation process, whereas PINK1ΔMTS simply accumulates in the cytoplasm. These co-IP data indicate that Clu can form a complex, either directly or indirectly, with PINK1 under normal cell culture conditions, and form a complex with Park at the mitochondrial outer membrane only. Furthermore, because Clu can interact with PINK1ΔMTS in the cytoplasm, the Clu-PINK1 interaction is independent of TOM20 or other mitochondrial outer-membrane proteins.

### Lack of Clu decreases mitochondrial protein levels and causes PINK1 and Park to interact

**Fig. 7.**
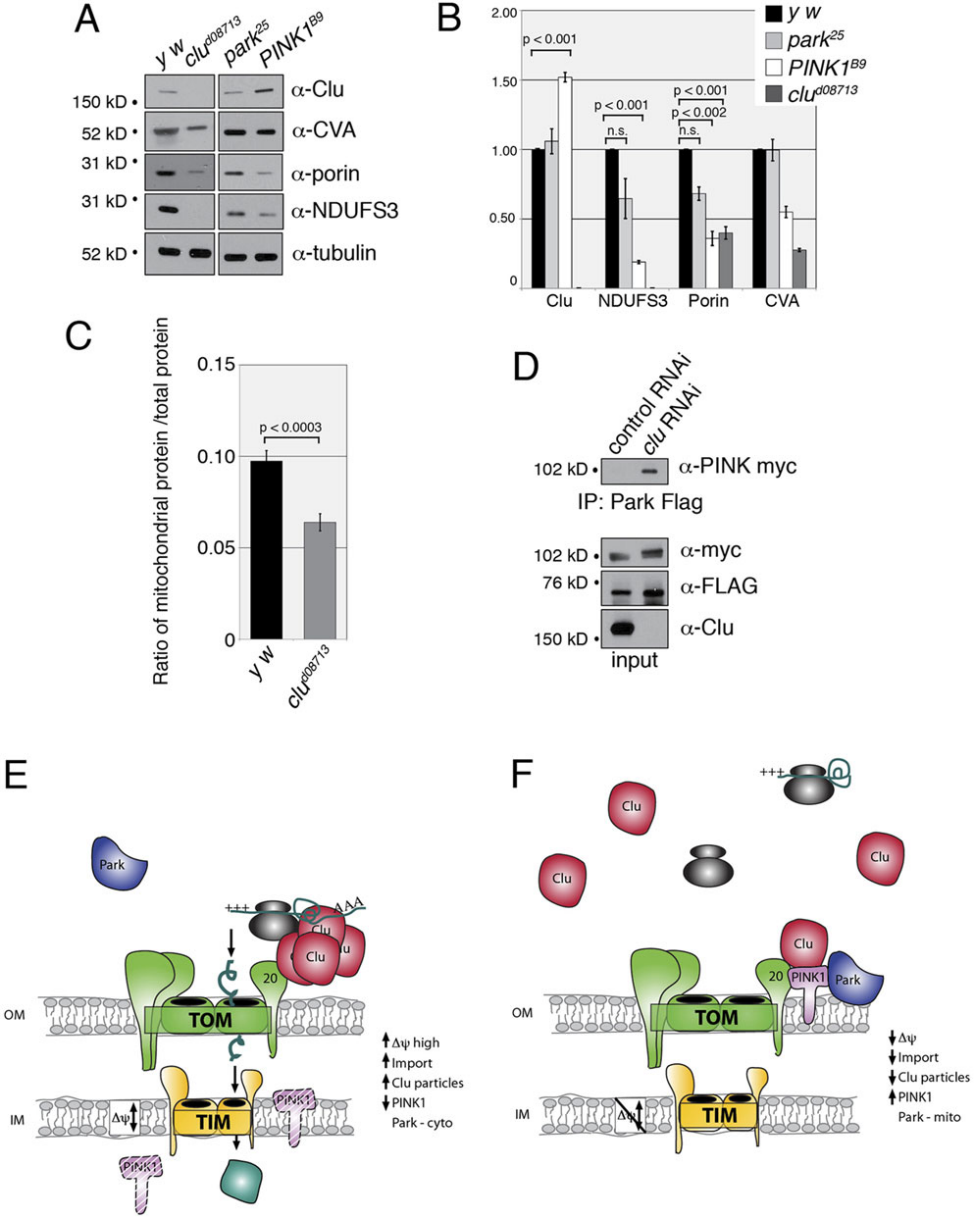
**Lack of Clu decreases mitochondrial protein levels and causes PINK1 and Park to interact.** (A) A representative western blot showing that levels of mitochondrial proteins are reduced in *clu* and *PINK1^B9^* mutant adults, but are not substantially reduced in *park* mutants. (B) Quantification of mitochondrial proteins in *park*, *PINK1^B9^* and *clu* mutant adults. Protein levels were normalized to tubulin for a loading control, then normalized to wild type (*y w*) for comparison. The error bars represent ±s.e.m. *P*-values were determined using Excel 2004 Toolpak and a two-tailed Student's *t*-test for each comparison. (C) In *clu* mutants, the ratio of mitochondrial protein to total protein is significantly smaller compared with wild type (*y w*). (D) PINK1-myc and Park-FLAG co-transfected S2R+ cells treated with control (luciferase) or *clu* RNAi show that PINK1 and Park do not form a complex under normal cell culture conditions in the presence of endogenous Clu (control RNAi lane), but are able to co-immunoprecipitate in the absence of Clu (*clu* RNAi lane). (E,F) A working model for Clu function. (E) Healthy mitochondria have associated Clu particles, undergo normal mitochondrial protein import and have low levels of PINK1. (F) When protein import is disrupted, Clu particles are lost, PINK1 is stabilized on the outer mitochondrial membrane and is thus able to recruit Park. Clu may or may not directly bind TOM20 or PINK1.

Once mutant *Drosophila* reach adulthood, they have survived over many days and stages of development. Thus, the level of mitochondrial proteins in adults containing mutations in genes important for mitochondrial function will reflect compensatory cellular mechanisms that take place during development. Mitochondrial protein levels have been shown to be higher in *park* mutants compared with wild type, particularly those that function in the respiratory complexes, mostly Complex I, supporting that Park functions in normal protein turnover (Vincow et al., 2013). Whereas *PINK1* mutants also affect turnover of respiratory complex proteins, general mitochondrial protein turnover is unaffected (Vincow et al*.*, 2013). To investigate how loss of Clu affects mitochondrial protein levels and potential turnover, we examined mutants that were null for *park*, *PINK1* or *clu* to compare the relative amounts of several mitochondrial proteins. The proteins we assayed were: Clu; Complex V/ATP synthase (CVA); Porin; and NADH ubiquinone iron-sulfur protein 3 (NDUFS3), which is a component of Complex I. All four proteins are encoded by the nuclear genome. We used α-tubulin to normalize the amount of total cell extract used. Whereas *park*-null flies had normal levels of Clu, *PINK1* mutants showed an increase in Clu protein (Fig. 7A,B). *clu* and *PINK1* mutants both had decreased levels of Porin and CVA, with *clu* mutants being the generally more severe of the two (Fig. 7A,B). NDUFS3 was undetectable in *clu* mutants, but detectable at very low levels in *PINK1* mutants. *clu* mutants were also much sicker than either *PINK1* or *park* mutant flies, living only 3-4 days post-eclosion, compared to 4 weeks or longer for *PINK1* and *park* mutants (Clark et al., 2006; Greene et al., 2003; Sen et al., 2013; Yang et al., 2006). *park* mutants did not show a significant reduction in any of the proteins measured. These results show that *clu* and *PINK1* mutants have fewer mitochondrial proteins, and that *PINK1* mutant flies have increased levels of Clu even though they lack Clu particles. On the other hand, *park* mutants do not have a reduction in the same mitochondrial proteins. Only a limited number of antibodies are available for *Drosophila* mitochondrial proteins; therefore, we compared the total amount of mitochondrial protein via Bradford assay and found that *clu* mutants contain a reduced overall amount of mitochondrial protein compared with wild-type controls (Fig. 7C). These results could be due to fewer overall mitochondria in the fly's body, or to defects in mitochondrial turnover.

There is evidence that, upon mitochondrial membrane depolarization, PINK1 interacts with and directly phosphorylates Park (Kondapalli et al., 2012; Shiba-Fukushima et al., 2012). The presence of PINK1 on the mitochondrial outer membrane is required to recruit Park to mitochondria. To test whether Clu is required for this interaction, and what role Clu might have in mitochondrial protein turnover, we treated S2R+ cells with either control or *clu-*RNAi, then simultaneously transfected the cells with myc-tagged PINK1 and FLAG-tagged Park. In agreement with previous reports, PINK1 and Park did not co-immunoprecipitate under normal cell culture conditions with the addition of control RNAi treatment (Fig. 7D). However, after *clu*-RNAi treatment, we found that PINK1 and Park can co-IP; thus, Clu is not required for this interaction (Fig. 7D). Significantly, the absence of Clu in and of itself triggers the interaction between PINK1 and Park, and this happens without the addition of CCCP. This result supports the idea that Clu function is necessary to keep the PINK1-Park interaction from being triggered, thus effectively functioning as a negative regulator. Clu function might be directly involved in mitophagy; however, it is more likely that it is important for overall mitochondrial function (see Discussion).

## DISCUSSION

*Drosophila* Clu is a large, highly conserved protein that shares its Clu and TPR domains with its human homolog, CLUH. Expressing CLUH in flies that are mutant for *clu* rescues the mutant phenotypes; thus, the human protein can use the fly machinery to fulfill the role of Clu. To date, all our evidence supports the idea that Clu has a role in mitochondrial function; however, it has been unclear how direct it is. In this study, using IPs we show that Clu can associate with three proteins located on the mitochondrial outer membrane, TOM20, Porin and PINK1. Thus, Clu is not only a cytoplasmic protein, but can also be a peripherally associated mitochondrial protein, supporting the idea that this highly conserved protein directly affects mitochondrial function.

*clu* mutants share many phenotypes with *park* and *PINK1* mutant flies, including flight muscle defects and sterility (Clark et al., 2006; Cox and Spradling, 2009; Greene et al., 2003; Park et al., 2006; Sen et al., 2013). We found that mitochondria are also mislocalized in *PINK1* mutant germ cells, similarly to *park* mutants, and form large knotted clumps that include circularized mitochondria, which is consistent with increased fusion events. Mitochondria in *clu* mutant germ cells, on the other hand, do not show any signs of changes in fission or fusion (Cox and Spradling, 2009). *clu* also genetically interacts with *PINK1* and *park*, with double heterozygotes having clumped mitochondria in germ cells and a loss of Clu particles, and double knockdown of *clu* with *PINK1* or *park* in flight muscle causing an increase in abnormal wing posture (Fig. 3) (Cox and Spradling, 2009). Park functions in a pathway with PINK1 to elicit a mitophagic response, and overexpressing *park* can rescue *PINK1* phenotypes in *Drosophila* (Clark et al*.*, 2006; Park et al*.*, 2006). Using S2R+ cells and *clu* RNAi knockdown, we found that overexpressing Park, but not PINK1, causes mitochondria to disperse. In adult flies, overexpressing full-length *clu* rescues the abnormal wing phenotype as well as mitochondrial phenotypes of *PINK1* mutants, and overexpressing full-length *clu* or *CLUH* in *PINK1*, but not *park*, mutants rescues their thoracic indentation (Fig. 5). These results place *clu* upstream of *park*, but downstream of *PINK1*. PINK1 stabilization on the mitochondrial outer membrane signals for Park to translocate to the organelle and subsequently ubiquitinate different proteins on the mitochondrial surface (Geisler et al., 2010; Lazarou et al., 2012; Narendra et al., 2010a). Thus, it is somewhat surprising in *Drosophila* that loss of PINK1 can be rescued by increased amounts of Park, and suggests that there might be additional roles that Park plays in the cell. Our data support the idea that an excess of Park overcomes deficits in mitochondrial function because it can rescue a loss of Clu as well. Mitochondrial clumping seems to be one of the responses to mitochondrial damage, in our system and in human tissue culture cells; thus, the dispersal upon Park overexpression in *clu*-RNAi-treated S2R+ cells is likely a sign of better mitochondrial health (Vives-Bauza et al*.*, 2010).

Here, we show that Clu reciprocally immunoprecipitates with overexpressed PINK1 under normal cell culture conditions. PINK1 has been shown to directly bind TOM20, and Clu can also form a complex with TOM20, suggesting that all three proteins are found in close proximity at the mitochondrial membrane. We show that Clu still immunoprecipitates with PINK1 when PINK1 is no longer targeted to the mitochondrial outer membrane (PINK1ΔMTS). This result indicates that Clu forms a complex with PINK1 independent of TOM20 or any other mitochondrial outer membrane proteins. Under normal conditions, PINK1 degradation happens so quickly that there are undetectable levels found at the outer mitochondrial membrane. Therefore, how is it possible that Clu is found in a complex with PINK1 in the absence of mitochondrial damage? It is likely that overexpressed PINK1 overwhelms the normal degradation process, thus becoming aberrantly stabilized at the outer mitochondrial membrane. Alternatively, it is possible that low levels of mitochondrial damage could account for the PINK1 being stabilized at the outer membrane, and then being able to interact with Clu.

Mitophagy ultimately leads to mitochondrial degradation in the lysosome. Currently, the literature involving Park and PINK1 uses mitochondrial protein levels as a read-out of mitophagy. However, recent data shows that different mitochondrial proteins have different half-lives, likely depending on what type of protein quality-control mechanism they use (Abeliovich et al*.*, 2013; Vincow et al*.*, 2013). Recent papers have examined protein half-life and found that *Drosophila* and yeast mitochondrial proteins, particularly those of Complex I in the case of flies, have increased half-lives when mitophagy proteins are missing. In addition, mitochondrial protein quality control does not always require destruction of the entire mitochondrion, but can selectively destroy certain proteins (Soubannier et al., 2012; Yoshii et al., 2011). For the mitochondrial proteins we examined, all were greatly reduced in *clu* and *PINK1* mutants, but not substantially altered in *park* mutants. This suggests that the turnover of the mitochondrial proteins we examined is more sensitive to the absence of *clu* and *PINK1* than *park*. In this study, we found that Park and PINK1 form a complex in the absence of Clu. Thus, Clu is not necessary for this interaction, and loss of Clu causes a PINK1-Park interaction. This, plus the fact that Clu can be found at the outer mitochondrial membrane in a complex with both PINK1 and Park, suggests that Clu can influence mitochondrial quality or function, perhaps by regulating mitochondrial protein levels.

Yeast Clu1p was identified as a component of the eukaryotic initiation factor 3 (eIF3) complex and as an mRNA-binding protein (Mitchell et al., 2012; Vornlocher et al., 1999). From our IP and mass spectrometry data, we have evidence that it can associate with the ribosome as well (A.S. and R.T.C., unpublished data). Although CCCP is commonly used to force mitophagy and mitochondrial protein turnover, this treatment might not mimic the more subtle damage and changes mitochondria likely face *in vivo*. Mitochondrial protein import, for example, requires an intact mitochondrial membrane potential. Given our data, it is possible that Clu could function in co-translational import of proteins, as well as act as a sensor to couple PINK1-Park complex activation to how well protein import occurs (Fig. 6E). This would help explain why we found that loss of Clu triggers a PINK1-Park interaction. In addition, Park and PINK1 directly interact with Porin and TOM20, respectively, placing them and Clu at the same place at the outer mitochondrial membrane. Recently, CLUH has been found to bind mRNAs for nuclear-encoded mitochondrial proteins, supporting a potential role in co-translational import (Gao et al., 2014). Further experiments are required to understand the precise relationship between Clu, TOM20, PINK1 and Park.

Mitochondria clearly undergo targeted destruction and require robust quality-control mechanisms, which are very active areas of investigation. PINK1 and Park's molecular mechanisms are particularly relevant to Parkinson's disease, given that inherited mutations in *PARK2* and *PINK1* can cause early-onset Parkinsonism. The molecular mechanisms that control mitophagy are becoming increasingly complex, involving membrane and cell biology; however, to date, the field has yet to visualize and understand the role of basal mitophagy levels *in vivo*. In the future, studying mitochondria and Clu function in *Drosophila* germ cells could allow us to better understand the role of mitochondrial protein turnover and quality control in the normal life cycle of tissues.

## MATERIALS AND METHODS

### Fly stocks

The following stocks were used for experiments: *w^1118^*; *UASp FL-clu*, *w^1118^*; *UASp CLUH, clueless^d08713^*/CyO Act GFP, *clueless^EP969^*/CyO KrGFP (Cox and Spradling, 2009), *parkin^1^*/TM3 Act GFP (Cha et al*.*, 2005), *parkin^25^*/TM6B (Greene et al*.*, 2003), *w^1118^*;* Mhc-GAL4, UAS-PINK1* RNAi (gift from Bingwei Lu, Stanford University, Palo Alto, CA), *w^1118^; UAS-parkin* (gift from Leo J. Pallanck, University of Washington, Seattle, WA), *w^1118^; UAS-Atg1* (gift from Thomas Neufeld, University of Minnesota, Minneapolis, MN), *Mhc-GAL4* (gift from Bingwei Lu), *w^1118^; UAS-PINK1* (gift from Bingwei Lu), and *w^1118^*;* park^25^* (gift from L.J. Pallanck). *Df*(3)*Pc-MK*/TM2, *PINK1^B9^*/FM7i (Park et al*.*, 2006), *Df*(1)*BSC535*/*FM7h*, *w^1118^; UAS-lacZ*, and *Dp*(1,3)*DC026* were obtained from the Bloomington Drosophila Stock Center. UAS-*parkin* RNAi (VDRC^KK104363^) *UAS-Atg1* RNAi (VDRC^GD16133^) and UAS-*clu* RNAi (VDRC^GD42138^ and VDRC^KK100709^) were all obtained from the Vienna Drosophila Resource Center. For wild type, *y^1^ w^67g23^* was used. To obtain *PINK1^B9^*/*Df*(1)*BSC535* females, heterozygous *PINK1^B9^*/FM7i virgins were crossed to *Dp*(1,3)*DC026* males. *PINK1^B9^*/Y; *Dp*(1,3)*DC026*/+ male progeny were then crossed to *Df*(1)*BSC535*/*FM7h* virgins. *w*^−^, non-FM7 females [*PINK1^B9^*/*Df*(1)*BSC535*] were then dissected. Flies were reared on standard cornmeal fly media at 22°C or 25°C.

### Transgenic flies and constructs

Full-length open reading frames were amplified from the following *Drosophila* expressed sequence tags (ESTs) (Drosophila Genomics Resource Center, Bloomington, IN): *clu* (RH51925), *park* (FI05213), *PINK1* (GH20931), *TOM20* (LD34461) and *porin* (GH11331). The *CLUH* gene was synthesized commercially by Genewiz, Inc., NJ. For transgenic flies, *clu* and *CLUH* were cloned into pPW (The Drosophila Gateway Collection, Carnegie Institution of Washington, Baltimore, MD) and commercially injected (Genetic Services, Inc., Cambridge, MA). For S2R+ tissue culture constructs, *park*, *PINK1*, *TOM20* and *porin* were cloned into pAWM containing a C-terminal myc tag. *park* was also cloned into pAWF containing a C-terminal FLAG tag (The Drosophila Gateway Collection, Carnegie Institution of Washington, Baltimore, MD).

### Negative geotaxis, egg laying, wing posture, and thoracic indentation experiments

Negative geotaxis was measured according to Ali et al*.* (2011) with 2-week-old adult flies. Egg-laying was done according to Drummond-Barbosa and Spradling (2001). Each measurement was done in triplicate. For abnormal-wing-posture analysis, flies were cultured at room temperature (RT). The F1 progeny were collected (10-20 flies per vial) immediately after eclosion and maintained for 2 weeks at 29°C (for RNAi crosses) and 5 days at RT (for overexpression crosses, done in triplicate, 100-140 flies per genotype). The abnormal-wing-posture penetrance was calculated as the percentage of flies with either held-up or drooped wing posture. For thoracic indentation quantification with RNAi, groups of ten newly eclosed progeny of each genotype were placed into separate vials with food and maintained at 25°C for 1 week, then scored under a light microscope and the wing phenotype was scored by eye. For RNAi experiments, at least 120 flies (40 from each vial: 20 males and 20 females) were used in replicates/triplicates. Statistical analysis was performed using two-tailed *t*-test in GraphPad software or Excel.

### Immunofluorescence and transmission electron microscopy

Fattened females were dissected at RT in Grace's Insect Medium (modified) (BioWhittaker, Lonza, Cologne, Germany). Ovaries were fixed for 20 min in 4% paraformaldehyde and 20 mM formic acid solution (Sigma) made in Grace's. Tissues were washed three times, 10 min each with antibody wash buffer (1× PBS:0.1% Triton X-100:1% BSA) and were incubated in primary antibody overnight at 4°C. They were then washed 3×10 min and incubated overnight at 4°C in secondary antibody. After washing 3×10 min, DAPI was added for 5 minutes then removed, then Vectashield (Vector Laboratories, Inc.) was added. Flight muscle was dissected away from the thorax in fix, then fixed for 30 min and processed as described above. For S2R+ immunostaining, cells were seeded onto a coverglass placed inside a well of a multiwell plate. Before staining, the coverglass was washed twice with 1× PBS then fixed and stained as for fly tissues. The following primary antibodies were used: guinea pig anti-Clu N-terminus (1:2000) (Cox and Spradling, 2009), mouse anti-Complex V (CVA) (1:1000, Mitosciences, Inc., cat.#MS507), 1B1 (1:200, Developmental Studies Hybridoma Bank, University of Iowa, Iowa City, IA), mouse anti-myc (Sigma). The following secondary antibodies were used: anti-mouse IgG_2b_ Alexa-Fluor-488, anti-mouse IgG_1_ Alexa-Fluor-568, anti-guinea pig Alexa-Fluor-488 and -568 (Molecular Probes, Invitrogen). Samples were imaged using a Zeiss 710 or Zeiss 700 confocal microscope and 63× Plan Apo NA 1.4 lens. TEM was performed as described previously (Cox and Spradling, 2003). Micrographs were collected using a JEOL JEM-1011 electron microscope.

### Western blotting and immunoprecipitations

For Western blotting, proteins were separated on 4-20% polyacrylamide gels using a standard SDS-PAGE protocol. After electrophoresis, proteins were transferred onto a Hybond-ECL nitrocellulose membrane (GE Healthsciences, Inc.) then soaked with Ponceau S for 10 min and rinsed as a further loading control. Blots were exposed to the following antibodies: anti-Clu (1:15,000) (Cox and Spradling, 2009), anti-eIF3x (CLUH, 1:1000, Bethyl Labs), anti-TOM20 (1:2000, Santa Cruz), anti-pyruvate dehydrogenase (Mitosciences, Inc.), anti-α-tubulin (1:5000, Developmental Studies Hybridoma Bank, University of Iowa, Iowa City, IA), anti-Myc (1:5000, Sigma), anti-NDUFS3 (1:2000, Mitosciences, Inc.), anti-Porin (1:2000, Mitosciences, Inc.), anti-CVA (1:100,000, Mitosciences, Inc.), anti-FLAG (1:10,000, Sigma), anti-actin JLA20 (1:750, Developmental Studies Hybridoma Bank, University of Iowa, Iowa City, IA). For co-immunoprecipitation, cells were transfected using Effectene transfection reagent (Qiagen). Myc-tagged plant glucuronidase (GUS) was transfected and used as a negative control for Clu and myc IPs. If CCCP treatment was needed, 10 µM was added after 36 h of transfection. S2R+ cells were lysed in IP buffer [20 mM HEPES, pH 7.4; 50 mM KCl, 0.02% Triton X-100, 1% NP-40 (sub), 1 mM EDTA, 0.5 mM EGTA, 5% glycerol] supplemented with 1 mM DTT and Protease inhibitor cocktail (Roche) just before use. Extract was centrifuged at 12,000 ***g*** for 10 min and the supernatant was nutated with the appropriate antibodies at 4°C overnight. Protein AG magnetic beads were added to the tube, which were incubated for an additional 2 h. The complexes were separated on a magnetic rack and eluted in 1× Laemmli buffer. High-speed centrifugation co-IPs were performed as above, except 1% Triton X-100 was added to the IP buffer and the extract was spun at 135,000 ***g*** for 30 min. For quantification, each experiment was repeated in triplicate and the band intensities on the western blots were quantified using ImageJ (NIH) (Schneider et al*.*, 2012).

### Cell fractionation

Mitochondria were isolated using the Thomas Scientific Mitochondrial Isolation kit (cat. #89874) following the manufacturer's protocol with the following modifications: 5×10^6^ S2R+ cells were suspended in 80 µl of Reagent A supplemented with 1 µl of Reagent B and incubated for 5 min with occasional short vortexing. 80 µl of Reagent C was added before the mixture was spun at 700 ***g*** for 5 min. The supernatant was spun again at 1500 ***g*** for 5 min. Crude extract was collected before spinning the supernatant for a final spin at 12,000 ***g*** for 10 min. The crude mitochondrial pellet was rinsed twice with Reagent C, then lysed in 1× SDS sample buffer for western blotting. Mitochondria from ovaries were isolated in the same way as above, except that they were homogenized with Reagent A using a blue pestle and eppendorf tube. For total protein and mitochondrial protein measurements, adult flies were homogenized in Reagent A supplemented with Reagent B using a blue pestle along with 0.1 mm glass beads. Mitochondrial proteins were dissolved in 1/4 volume of the total volume. Protein levels were quantified using standard Bradford assay. The assay was performed in experimental duplicate and technical quadruplicate.

### S2R+ cell RNAi

*Drosophila* S2R+ cells were grown in Schneider's *Drosophila* media (Gibco) supplemented with 10% fetal bovine serum and 0.5% Penstrep. *clu* dsRNA was made from the 3′ UTR using primers 5′-TAATACGACTCACTATAGGGAGAACGCTCCCAATGGGCGATG-3′ and 5′-TAATACGACTCACTATAGGGAGACGGACGTGTCTGGTGATCCCG-3′. 1×10^5^ cells were seeded per well in a 48-well plate and the cells were grown in media containing 25 µM dsRNA for 4 days, with an additional 25 µM dsRNA added on the second day. RNAi-treated cells were transfected using Effectene transfection reagent (Qiagen) following the supplier's protocol. Mitochondrial clumping was documented after confocal analysis by determining when the majority of mitochondria form one to three clumps in an arbitrary quarter of a cell. For Figs 1B-E and 4A-L, each experiment was performed in technical triplicate.

## Supplementary Material

Supplementary Material
