# Supplementary material for: Clueless, a protein required for mitochondrial function, interacts with the PINK1-Parkin complex in Drosophila
Source: Dis Model Mech. 2015 Jun 1;8(6):577–89. doi: 10.1242/dmm.019208 (PMC4457034; doi:10.1242/dmm.019208)
Supplement: Supplementary Material [file supp_8_6_577__index.html]

Clueless, a protein required for mitochondrial function, interacts with the PINK1-Parkin complex in Drosophila — Supplementary Material 

# Clueless, a protein required for mitochondrial function, interacts with the PINK1-Parkin complex in *Drosophila*

## DMM019208 Supplementary Material

**Files in this Data Supplement:**

- **Supplementary Material**
