# Supplementary material for: Clueless, a protein required for mitochondrial function, interacts with the PINK1-Parkin complex in Drosophila
Source: Dis Model Mech. 2015 Jun 1;8(6):577–89. doi: 10.1242/dmm.019208 (PMC4457034; doi:10.1242/dmm.019208)
Supplement: Supplementary Material [file supp_8.6.577_DMM019208.pdf]

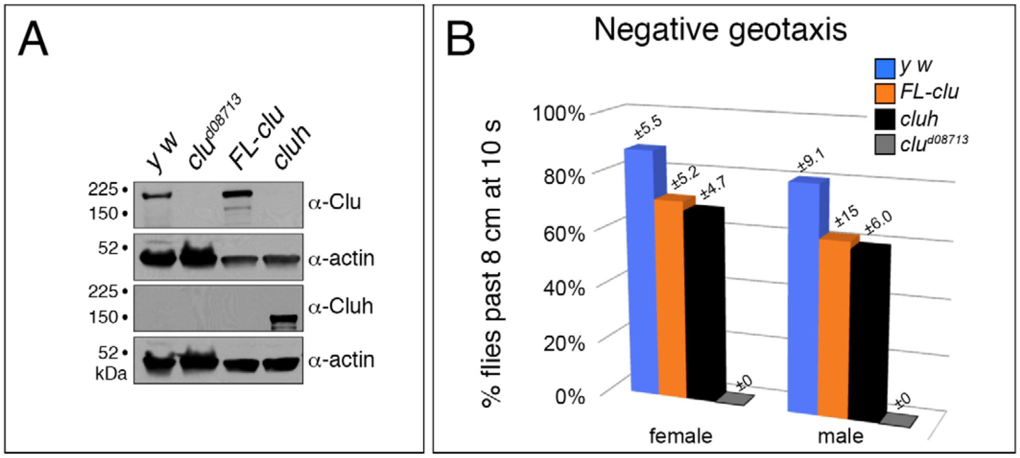

Figure S1. Cluh expression rescues *clu* mutant flies. (A) Western blot showing Clu and Cluh expression in transgenic flies expressing full length *clu* (*FL-clu*) and *cluh* under the control of UASp driven by ubiquitous *da*-GAL4 in a *clu*^{d08713} background. (B) *UASp-FL-clu* and *UASp-cluh* rescue the *clu*^{d08713} climbing defect to the same degree in males and females. Both transgenes were expressed using *da*-GAL4.

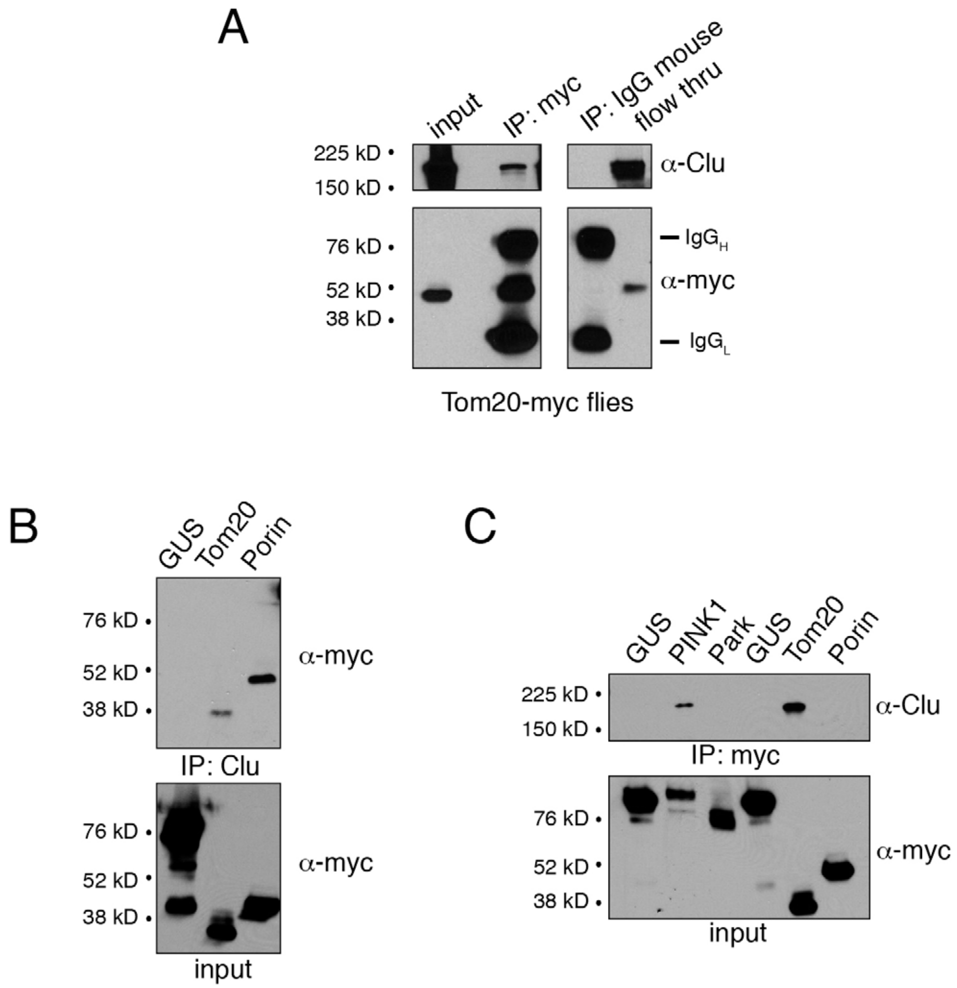

Figure S2. TOM20 and Porin form a complex with Clu. (A) Extract from flies overexpressing UAS-TOM20-myc using ubiquitous ActGAL4. Using myc antibody for the IP shows Clu is present. Mouse IgG was used for the IP as a negative control. Neither Clu nor TOM20 is present in the IgG IP, but both are present in the flow thru. (B, C) Co-immunoprecipitation after high-speed centrifugation. S2R+ cell extract was treated with detergent followed by high-speed centrifugation to ensure mitochondrial outer membrane proteins are solubilized. (A) Using Clu antibody to IP, and myc antibody to probe shows TOM20 and Porin interact with Clu. (B) Performing the reverse IP, using myc antibody to immunoprecipitate and Clu antibody to probe, confirms the Clu-TOM20 interaction, but not the Clu-Porin interaction. GUS = myc tagged plant glucuronidase was tranfected and subjected to IP with myc antibody as a negative control.

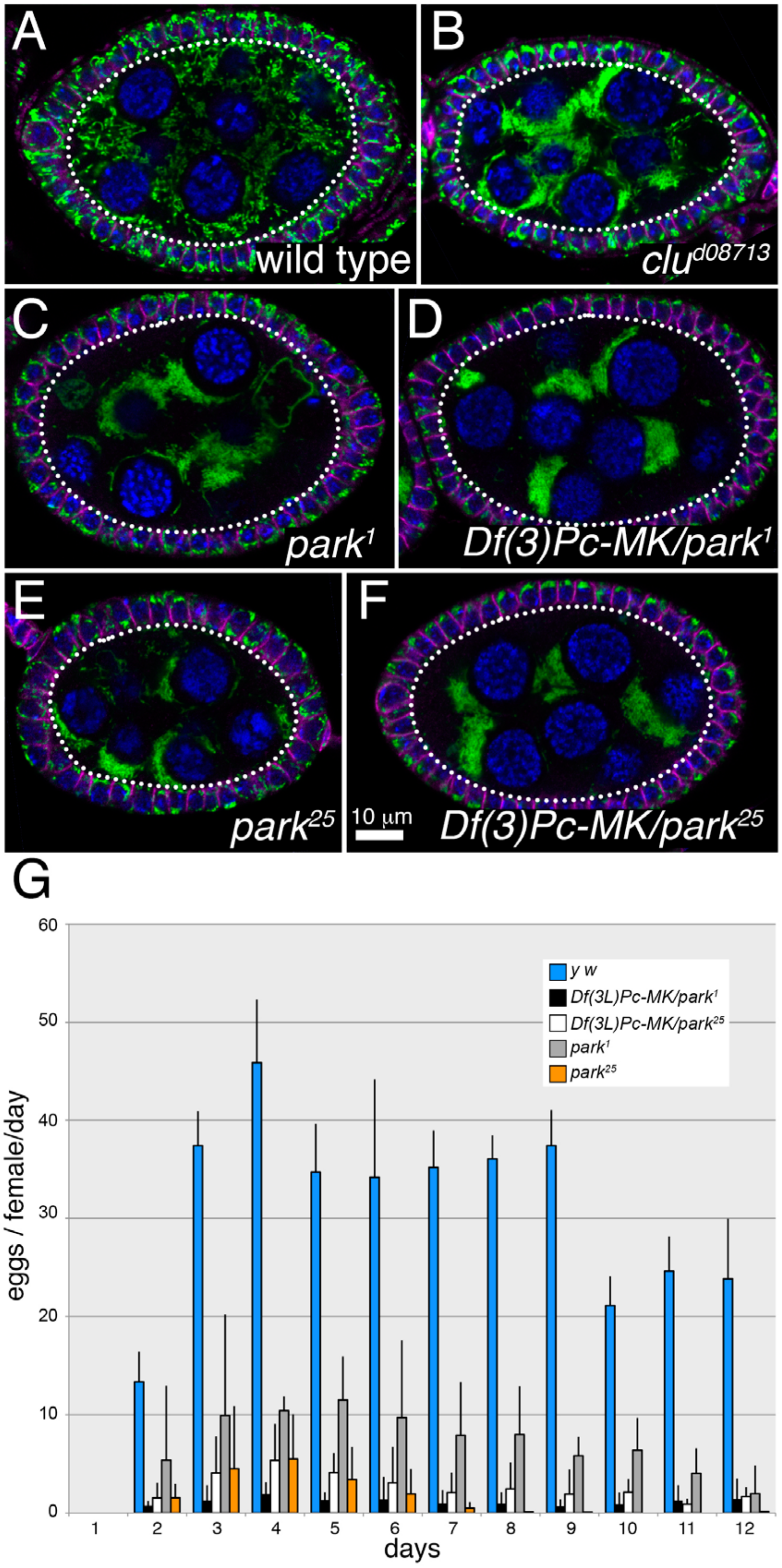

Figure S3. *park* mutant flies have greatly reduced fertility. (A-F) Mitochondrial distribution in germ cells. (A) A wild type follicle showing evenly distributed mitochondria (green) in the germplasm (dotted line). Somatic follicle cells are indicated by magenta. (B) A clu^{d08713} mutant follicle showing characteristic mitochondrial clumping. (C-F) *park* mutant follicles also exhibit severe mitochondrial clumping. Two previously independently generated null alleles, $park^{25}$ and $park^{1}$ have identical mitochondrial clumping phenotypes (C, E), including when they are hemizygous over the deficiency *Df(3)Pc-MK* (D, F). (G) All four *park* allelic combinations result in greatly reduced fertility as assayed by egg laying. $park^{1}$ lays the most eggs. As $park^{25}$ lays the fewest eggs, we used this allele for our experiments. Green = anti-CVA, magenta = anti-1B1, blue = DAPI (A-F). Scale bar = 10 mm in F for A-F.
